# Vulnerability of Walnut Pruning Wounds to Fungal Trunk Pathogens and Seasonal Conidial Dynamics of Botryosphaeriaceae in the Maule Region, Chile

**DOI:** 10.3390/microorganisms13102407

**Published:** 2025-10-21

**Authors:** Shehzad Iqbal, Iqra Mubeen, Mauricio Lolas, Ernesto Moya-Elizondo, Pedro Gundel, Samuel Ortega-Farias, William Campillay-Llanos, Gonzalo A. Díaz

**Affiliations:** 1 Laboratorio de Patología Frutal, Departamento de Producción Agrícola, Facultad de Ciencias Agrarias, Universidad de Talca, Campus Talca, Av. Lircay s/n, Talca 3460000, Chile; shehzad.iqbal@utalca.cl (S.I.); iqra.mubeen@utalca.cl (I.M.); mlolas@utalca.cl (M.L.); 2 Departamento de Producción Vegetal, Facultad de Agronomía, Universidad de Concepción, Chillán 3812120, Chile; emoya@udec.cl; 3 Centro de Ecología Integrativa, Instituto de Ciencias Biológicas, Universidad de Talca, Campus Talca, Av. Lircay s/n, Talca 3460000, Chile; pedro.gundel@utalca.cl; 4 Instituto de Biología Funcional y Biotecnología (BIOLAB)-INBIOTEC-CONICET-CICBA, Facultad de Agronomía, Universidad Nacional del Centro de la Provincia de Buenos Aires (UNICEN), Av. República de Italia # 780, Azul 7300, Buenos Aires, Argentina; 5 Centro de Investigación y Transferencia en Riego y Agroclimatología (CITRA), Universidad de Talca, Campus Talca, Av. Lircay s/n, Talca 3460000, Chile; sortega@utalca.cl; 6 Departamento de Ciencias Matemáticas y Físicas, Universidad Católica de Temuco, Rudecindo Ortega 02950, Región de la Araucanía, Temuco 4813302, Chile; wcampillay@uct.cl; 7 Nucleo de Investigación en Producción Alimentaria, Facultad de Recursos Naturales, Universidad Católica de Temuco, Temuco 4813302, Chile

**Keywords:** airborne inoculum, Botryosphaeriaceae, *Diaporthe*, *Neofusicoccum nonquaesitum*

## Abstract

Branch canker and dieback, caused by Botryosphaeriaceae and Diaporthaceae, is a major disease of walnut (*Juglans regia* L.) worldwide. In Chile, the impact of pruning wound age and timing on susceptibility to these pathogens in walnut trees remains poorly understood. During June–July (2023) and June–July (2024), this study assessed the effect of pruning wound age of the walnut cv. Chandler on infection by seven fungal species and simultaneously tracked seasonal conidial release of Botryosphaeriaceae spp. in the Maule Region, Chile. Lignified twigs were artificially inoculated at 1, 15, 30, and 45 days after pruning, and necrotic lesion lengths were measured six months post-inoculation. All fungal isolates caused significantly longer lesions than the control (*p* < 0.0001), with *Diplodia mutila*, *Neofusicoccum nonquaesitum*, and *N. parvum* being the most aggressive. At the same time, *Dothiorella sarmentorum* and *Diaporthe* species (*Diaporthe australafricana*, *Di. foeniculina*, and *Di. patagonica*) produced the smallest lesions. Susceptibility decreased with increasing wound age, with a significant interaction between fungal species and pruning wound age. Spore trapping of Botryosphaeriaceae revealed that dispersal was positively associated with rainfall (*r* = 0.81, *p* < 0.0001), relative humidity (*r* = 0.51 to 0.61, *p* < 0.05) and average temperature (*r* = 0.32 to 0.58, *p* < 0.05), but negatively or not significantly correlated with maximum temperature (*r* = −0.59 to −0.79, *p* > 0.05). These results demonstrate that rainfall or relative humidity, moderate conditions, and favor conidial release. At the same time, infection risk declines with wound age, underscoring the need to adjust pruning schedules and preventive strategies to reduce disease risk in walnut orchards.

## 1. Introduction

The English walnut (*Juglans regia* L.) is a globally significant nut crop, highly valued for its nutritional properties and economic impact. As a deciduous species, walnut trees are cultivated commercially across diverse regions, with global production reaching approximately 2.67 million metric tons in the 2024/2025 season. Walnut cultivation is concentrated in a few key producing countries: China leads with 56% of global output (around 1.5 million metric tons), followed by the United States at 23% (608,000 metric tons), Chile at 7% (195,000 metric tons), and the European Union collectively contributing 6% (150,000 metric tons) [1]. Chile’s walnut industry exemplifies the crop’s economic relevance, with trade revenues reaching $327 million in 2024—a 32% increase from the previous year. The average export price of $2894 per ton in 2022 further underscores the species’ market adaptability and high commercial value across varied geographic and climatic conditions [2].

Fungal pathogens belonging to the families Botryosphaeriaceae and Diaporthaceae are recognized as primary causal agents of branch canker and dieback in walnut trees [3,4,5,6]. These trunk-infecting fungi produce characteristic elliptical cankers with brown to orange bark discoloration, often leading to the death of fruiting limbs [6]. Infected wood typically shows internal necrosis ranging from brown to dark brown, accompanied by a marked increase in hardness. Cross-sectional analysis typically reveals V- or U-shaped necrotic patterns, while longitudinal cuts expose continuous brown streaks along the vascular tissue [7]. These symptoms underscore the destructive potential of these pathogens and highlight the need for effective diagnostic and management strategies in walnut cultivation.

Studies indicate that the prevalence of branch canker and dieback on walnut ranges significantly, with disease incidences reported between 40 and 60% in regions such as Australia [8], California [3], China [9], France [10], Iran [11], Italy [12], Spain [6], the Czech Republic [5], and Turkey [13]. In Chile, the incidence of canker and dieback diseases varies between 10% and 40%, depending on the orchard management practices and environmental conditions [14,15,16,17,18,19]. However, comprehensive reports on the incidence and prevalence of dieback diseases in English walnut throughout Chile remain limited, with available data primarily concentrated in central Chile regions.

Currently, several species of the family Botryosphaeriaceae have been associated with branch canker and dieback of walnut and other woody plants worldwide, including almond [20], apple [21], avocado [22], blueberry [23], European hazelnut [24,25,26], English walnut [3], grapevine [27,28], olive [29] and pistachio [30]. These host fruit trees are affected by species of the genera *Botryosphaeria*, *Diplodia*, *Dothiorella*, *Lasiodiplodia*, *Neofusicoccum*, and *Neoscytallidium* [3,31,32,33]. Similarly, the family Diaporthaceae, represented by Diaporthe species with more than 200 species reported worldwide [34], also cause canker and dieback diseases across various hosts including apple [35], blueberry [36], citrus [37], European hazelnut [38], grapevine [39], kiwifruit [40,41], olive [29] and walnut [42]. This taxonomic diversity emphasizes the complex etiology of walnut branch canker and dieback and highlights the necessity of vigilant monitoring to comprehend the epidemiology and impacts of this disease on walnut production [6].

Moreover, several species of Botryosphaeriaceae and Diaporthaceae have also been identified as causal agents of severe losses on branches, shoots, and fruit diseases in Chilean walnut orchards, resulting in severe economic losses through significant reductions in fruit production, such as *Diplodia mutila* [14], *D. seriata*, *Neofusicoccum parvum*, *Diaporthe australafricana*, and *Di. cynaroidis* [15,16], *Neofusicoccum australe* [17], *Dothiorella sarmentorum* [18], and *N. nonquaesitum* [19].

Epidemiologically, the release and dissemination of airborne spores (mainly conidia) produced by Botryosphaeriaceae are crucial steps for understanding the infection processes of branch canker and dieback on walnut in the field [7,43]. Release and distribution of airborne spores of Botryosphaeriaceae influenced by environmental factors have been studied on apple [44], avocado [45], English walnut [43], and grapevine [46]. Furthermore, Moral et al. [7] suggested that the highest levels of conidial release and subsequent infection occur during wet periods in the autumn and winter under Mediterranean climate conditions in walnut. However, information on the seasonal dynamics of Botryosphaeriaceae spore release in English walnut orchards is scarce and null in Chile.

Pruning is a widely adopted cultural practice in walnut orchards, primarily aimed at optimizing canopy architecture, enhancing light penetration, and reducing pest pressure. However, this practice also introduces epidemiological risks. Fresh pruning wounds act as primary infection courts for conidia of fungal trunk pathogens, which are predominantly dispersed by wind and rain splash—two key environmental factors that facilitate pathogen entry [7,47]. The vulnerability of fresh pruning wounds to pathogen invasion has been documented globally across various fruit and nut crops, including almond [48], apple [44,49], grapevine [27,50,51], and English walnut [4,52,53]. Several studies have demonstrated that susceptibility decreases significantly over time in grapevine [50,54], apple [44], and walnut [52]. However, research on Botryosphaeriaceae and Diaporthaceae infection via pruning wounds is scarce in crop systems, resulting in a knowledge gap regarding these pathogen groups.

The contribution of this study is to address existing gaps in the understanding of walnut disease dynamics. Thus, the objectives were (i) to evaluate how the age of pruning wounds affects lesion development on lignified walnut twigs inoculated with species from the Botryosphaeriaceae and Diaporthaceae families; and (ii) to monitor the seasonal dispersal of Botryosphaeriaceae spp. fungal spores under orchard conditions in the Maule Region, correlating spore release and weather variables to identify periods of elevated infection risk. The findings will contribute valuable insights into the epidemiology of walnut diseases and support the development of effective, science-based management strategies.

## 2. Materials and Methods

### 2.1. Experimental Site

The experiments were performed during two consecutive seasons (2023 and 2024) on 12-year-old mature walnut (*Juglans regia*) trees cv. Chandler. The effect of pruning wound age on lesion development caused by Botryosphaeriaceae and Diaporthaceae was evaluated on one-year-old lignified twigs in a commercial orchard located in San Rafael (35°23′3″ S, 71°26′42″ W), Maule region, Chile. This orchard is situated at 100 m above sea level. A temperate Mediterranean climate (Csb; Köppen–Geiger climate classification) is characterized by cool, wet winters and dry, warm summers (www.meteochile.cl, accessed on 1 January 2023). Trees were trained under a central-leader system, without support structures, and planted at a typical Chilean spacing of 5 m between rows and 7 m between trees, balancing productivity and orchard management efficiency. The (0.5-ha) orchard was managed under an Integrated Pest Management (IPM) program with a reduced pesticide application, and no fungicides were applied from April to September in both seasons.

### 2.2. Fungal Isolation and Inoculum Preparation

The four Botryosphaeriaceae isolates studied included *D. mutila* (SC-CH-1), *Do. sarmentorum* (Dsar-1), *N. nonquaesitum* (VLC-1-6-20), and *N. parvum* (VLC-1-1-20). In addition, three species of the Diaporthaceae family, *Di. australafricana* (VLC-1-2-20), *Di. foeniculina* (SR-8), and *Di. patagonica* (VLC-2-5-20), were previously obtained from branch dieback samples in the Maule region, Chile [18,19]. The fungal isolates were purified and maintained on potato dextrose agar (PDA) in a Petri dish at 4 °C in the fungal collection of the Fruit Pathology Laboratory, University of Talca, until use.

Each isolate was reactivated for inoculation by transferring a 5 mm mycelium plug to fresh acidified potato dextrose agar (APDA) [prepared by adding 0.5 mL L^−1^ of 92% lactic acid to PDA] and incubated at 25 ± 2 °C for 10 days. Mycelial suspensions were then prepared as follows: (i) cultured mycelium was scraped and homogenized in sterile distilled water using a blender; (ii) the resulting mixture was filtered through two layers of cheesecloth and stirred; (iii) one drop of Tween 20 (Sigma-Aldrich, Darmstadt, Germany) was added; and (iv) the concentration of each fungal suspension was adjusted to 3 × 10^5^ mycelial fragments/ml using a hemocytometer [55]. Suspensions were freshly prepared 12 h before inoculation and maintained at 4 °C until applied.

### 2.3. Susceptibility of Pruning Wound Age to Fungal Trunk Pathogen Infections

Field experiments were carried out in June–July (2023) and June–July (2024) in a commercial walnut orchard in San Rafael, Chile, to investigate pruning wound susceptibility to Botryosphaeriaceae and Diaporthaceae species in the walnut trees cv. Chandler, considering the age of the wound after pruning. Seven species representing seven isolates of pathogens were employed. A single isolate for each species was chosen for the purpose of experimentation. Healthy, lignified dormant twigs (20–30 cm long) from 7 to 15 year old trees, each with seven buds, were selected for the study. Twigs were pruned manually using sterilized INOX Felco pruning shears (La Horqueta, Santiago, Chile). Wounds were inoculated at 1, 15, 30, and 45 days after pruning with 100 μL of a mycelial suspension applied directly onto each wound using a micropipette. To prevent rapid desiccation, inoculated wounds were immediately wrapped with parafilm tape.

Pruning wounds were slightly moistened with sterile distilled water before inoculation to simulate natural conditions and facilitate mycelium fragment adherence. Twigs were then left under field conditions for six months. During this period, natural environmental factors, such as rainfall, humidity, and temperature, were collected. After six months, necrotic lesion development was evaluated by measuring the length of tissue discoloration extending downward from each pruning wound (in millimeters). To fulfill Koch’s postulates, small fragments of necrotic tissue were collected from the edges of lesions, plated onto APDA, and incubated. Re-isolated fungi were identified based on colony characteristics and conidial morphology according to previous authors [21,31,32].

### 2.4. Spore Trapping and Monitoring

To assess the spore dispersal of Botryosphaeriaceae species, because preliminary surveys revealed these fungi were the major pathogens of walnut trees in the commercial orchards of the Maule region, Chile. Spore traps were prepared using glass microscope slides (25 × 76 mm) coated with a thin layer of white Vaseline on both sides [56]. Slides were attached with clips to a 7- to 15-year-old cv. Chandler walnut trees in an orchard with Botryosphaeria-related branch canker and dieback symptoms. Slides were positioned approximately 1.5 m above the ground. A total of 20 slides were randomly distributed across 10 tree rows, with each row containing 40 trees. Traps were replaced every two weeks throughout the 2023 and 2024 growing seasons and transported to the Fruit Pathology Laboratory at the University of Talca for processing.

Conidia adhering to the slides were collected by placing each slide in a sterile 50 mL Falcon tube containing 10 mL of distilled water at room temperature and gently agitating by hand for 60 s. Aliquots of 200 μL were taken from each tube, stained with 50% lactophenol blue (Merck KGaA, Darmstadt, Germany), and examined under a Nikon optical microscope at 40× magnification (Nikon Solutions Co., Ltd., Tokyo, Japan). Each conidium observed was characterized based on morphological features, including shape, size, color, and septation. Morphological data were compared with previously published descriptions [21,31,32] to determine genus and species identification. Conidia counts were recorded for the entire surface area of each slide, with results expressed as the total number of conidia collected per two-week interval. All 20 slides were examined for each sampling event, and the data were systematically recorded.

### 2.5. Weather Variables and Data Analysis

Weather and spore data were collected to evaluate the relationship between environmental conditions and the release of Botryosphaeriaceae conidia in walnut orchards. Meteorological data included daily average and hourly temperature, relative humidity, wind speed, total daily precipitation, and total hours of precipitation per day. In the Maule region of Chile, meteorological data were collected from stations adjacent to walnut orchards in the Pelarco area (near San Rafael) through the computer platform of the Agroclimatological Network of the Institute of Agricultural Research INIA (https://agrometeorologia.cl/ accessed on 1 January 2023). Weekly meteorological values were calculated by summing daily rainfall and averaging daily temperature and relative humidity.

Pearson correlation analyses were conducted to examine the relationships between environmental variables and the release of Botryosphaeriaceae conidia. The total number of conidia was correlated with weekly averages of environmental variables, including accumulated rainfall and relative humidity, using SigmaStat version 4.0 (Systat Software, Inc. Chicago, IL, USA). These analyses aimed to determine how temperature, humidity, rainfall, and other meteorological factors influence the seasonal release and dispersal of Botryosphaeriaceae spores in walnut orchards.

### 2.6. Experimental Setup and Statistical Analysis

Field experiments were conducted on walnut trees to evaluate infection caused by Botryosphaeriaceae and Diaporthaceae species. Treatments were arranged in a completely randomized design, with three replicates per treatment. Each experimental unit consists of seven healthy and lignified twigs per tree. A control treatment per pathogen species was considered, but this was excluded from statistical analyses, and it was used only to estimate the potential natural infection associated with the ambient inoculum. Percent of wounds infected (%) was analyzed as a two-way factorial (fungal species × age of pruning wounds). Data were arcsine square root-transformed before analysis to meet assumptions of normality. Analysis of variance (ANOVA) was performed, and treatment means were compared using Fisher’s Least Significant Difference (LSD) test at *p* < 0.05. Data analysis was conducted in R Studio version 4.1.0.

## 3. Results

### 3.1. Pruning Wound Age on Infection by Botryosphaeriaceae and Diaporthaceae spp. in English Walnut

All pruning wounds on artificially inoculated branches developed lesions after incubating in the 2023 and 2024 experimental trials. The analysis of variance revealed highly significant effects (*p* < 0.0001) of pruning wound age, fungal species, and their interaction on lesion development in both experimental years. The mean lesion length varied significantly among fungal isolates and decreased progressively with increasing wound age across all tested species. These results clearly demonstrate that both the specific pathogen and the age of the pruning wound are critical factors determining the success of infection in walnut trees.

**Figure 1:**
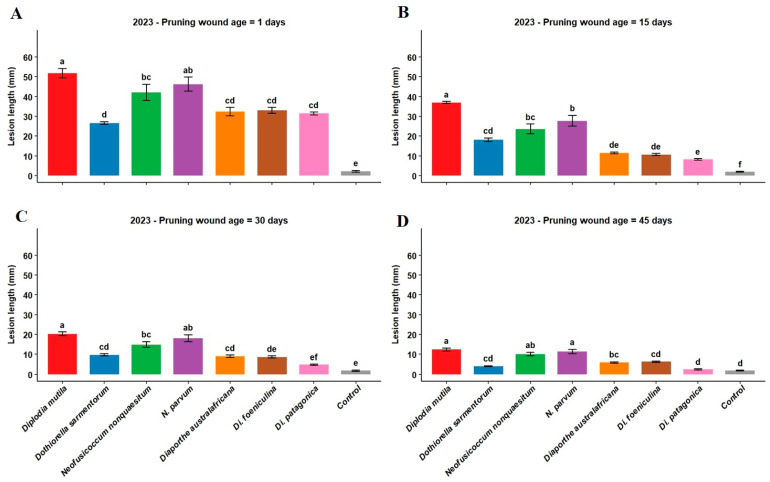
(**A**–**D**): Lesion length (mm) induced by *Diplodia mutila* (SC-CH-1, red), *Dothiorella sarmentorum* (Dsar-1, blue), *Neofusicoccum nonquaesitum* (VLC-1-6-20, green), *N. parvum* (NP-2022, purple), *Diaporthe australafricana* (VLC-1-2-20, orange), *Di. foeniculina* (SR-8, brown), and *Di. patagonica* (VLC-2-5-20, pink): control = sterile agar plug (gray), on pruning wounds of ‘Chandler’ English walnut of different ages; 1 day (**A**), 15 days (**B**), 30 days (**C**), and 45 days (**D**), during 2023. The lesions were measured at 6 months post-inoculation. Error bars represent mean lesion length, and letters above the bars imply significant differences by Fisher’s LSD test (*p* < 0.05).

In 2023, inoculation of fresh pruning wounds (1-day post-pruning) produced significantly more extensive lesions than older wounds across all fungal species tested (Figure 1A). *Diplodia mutila* (SC-CH-1) caused the most aggressive infections, producing lesions of 51.8 mm on 1-day-old wounds, followed by *N. parvum* (NP-2022) with 46.2 mm and *N. nonquaesitum* (VLC-1-6-20) with 42.1 mm. In contrast, *Do. sarmentorum* (Dsar-1), *Di. australafricana* (VLC-1-2-20), *Di. foeniculina* (SR-8), and *Di. patagonica* (VLC-2-5-20) produced significantly shorter lesions on fresh wounds (Figure 1A). Therefore, fresh wounds are universally susceptible, but the extent of infection is highly dependent on the pathogen species, with Botryosphaeriaceae members like *D. mutila* and *N. parvum* posing the most severe threat immediately after pruning.

The susceptibility of pruning wounds declined markedly with age. At 15 days post-pruning, lesion lengths decreased substantially. However, *D. mutila* maintained the highest pathogenicity with lesions of 36.9 mm, followed by *N. parvum* and *N. nonquaesitum* (Figure 1B). Wounds inoculated 30 and 45 days after pruning showed further reduced susceptibility across all fungal species (Figure 1C,D). Notably, *Di. patagonica* produced minimal lesions of only 2.4 mm on 45-day-old wounds, while *Do. sarmentorum* and other *Diaporthe* species exhibited consistently low pathogenicity across all wound ages. The control treatments developed only minor, non-progressive lesions, which served as the baseline for comparison. For the majority of pathogens, the resulting lesion lengths were significantly greater than those in the control (Figure 1A,B,D), confirming that wound inoculation was the primary driver of lesion development. While wound susceptibility decreases over time, the protection offered by wound aging is pathogen-specific, with certain Botryosphaeriaceae species remaining capable of causing significant lesions even on 15-day-old wounds.

**Figure 2:**
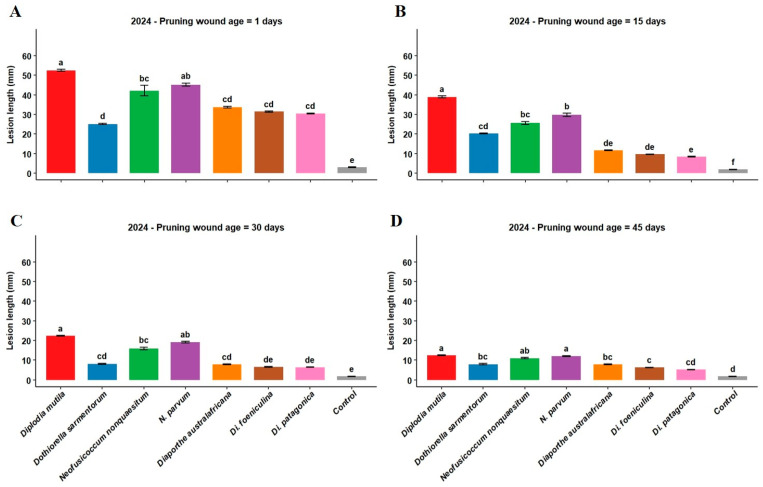
(**A**–**D**): Lesion length (mm) induced by *Diplodia mutila* (SC-CH-1, red), *Dothiorella sarmentorum* (Dsar-1, blue), *Neofusicoccum nonquaesitum* (VLC-1-6-20, green), *N. parvum* (NP-2022, purple), *Diaporthe australafricana* (VLC-1-2-20, orange), *Di. foeniculina* (SR-8, brown), and *Di. patagonica* (VLC-2-5-20, pink): control = sterile agar plug (gray), on pruning wounds of ‘Chandler’ English walnut of different ages; 1 day (**A**), 15 days (**B**), 30 days (**C**), and 45 days (**D**), during 2024. The lesions were measured at 6 months post-inoculation. Error bars represent mean lesion length, and letters above the bars imply significant differences by Fisher’s LSD test (*p* < 0.05).

The 2024 trial confirmed the trends observed in the previous year, with fresh wounds showing the highest susceptibility to infection (Figure 2). *Diplodia mutila* (SC-CH-1) remained the most aggressive pathogen, causing maximum lesion development of 52.5 mm on 1-day-old wounds, followed by *N. parvum* (45.2 mm) and *N. nonquaesitum* (42.1 mm) (Figure 2A). The progressive decline in wound susceptibility with age was consistent across all tested isolates.

Interestingly, some species showed year-to-year variability in their pathogenicity on aged wounds. *Dothiorella sarmentorum*, *Di. australafricana*, and *Di. foeniculina* caused relatively more extensive lesions on older wounds in 2024 compared to 2023, suggesting possible environmental or physiological factors influenced pathogen establishment and development. *Diaporthe patagonica* maintained low pathogenicity across both years, producing 6.3 mm and 5.8 mm lesions on 30 and 45-day-old wounds, respectively, in 2024 (Figure 2C,D). Collectively, these results confirm the consistent threat posed by key Botryosphaeriaceae species on fresh wounds across seasons, while also highlighting that the behavior of less aggressive pathogens can be influenced by inter-annual variables.

Analysis of variance revealed significant main effects for both fungal species and wound age (*p* < 0.0001) on lesion development. The interaction between fungal species × wound age was highly significant (*p* = 0.0001) in both experimental years, indicating that the pathogen response to wound age varied among fungal species. This interaction demonstrates that the temporal window of susceptibility differs among the tested pathogens, with some species maintaining pathogenicity on older wounds more effectively than others.

All Botryosphaeriaceae and Diaporthaceae isolates were successfully re-isolated from infected plant tissues on APDA medium and confirmed through morphological and molecular analyses, fulfilling Koch’s postulates. No fungal growth was observed in negative control treatments, confirming the specificity of the experimental infections. Therefore, the highly significant interaction effect not only confirms a statistically distinct model of infection but also biologically defines species-specific infection windows, which are critical for tailoring timely control strategies.

### 3.2. Monitoring of Botryosphaeriaceae Spore Dispersal and Weather Conditions

#### 3.2.1. Climate-Spore Relationship-2023

**Figure 3:**
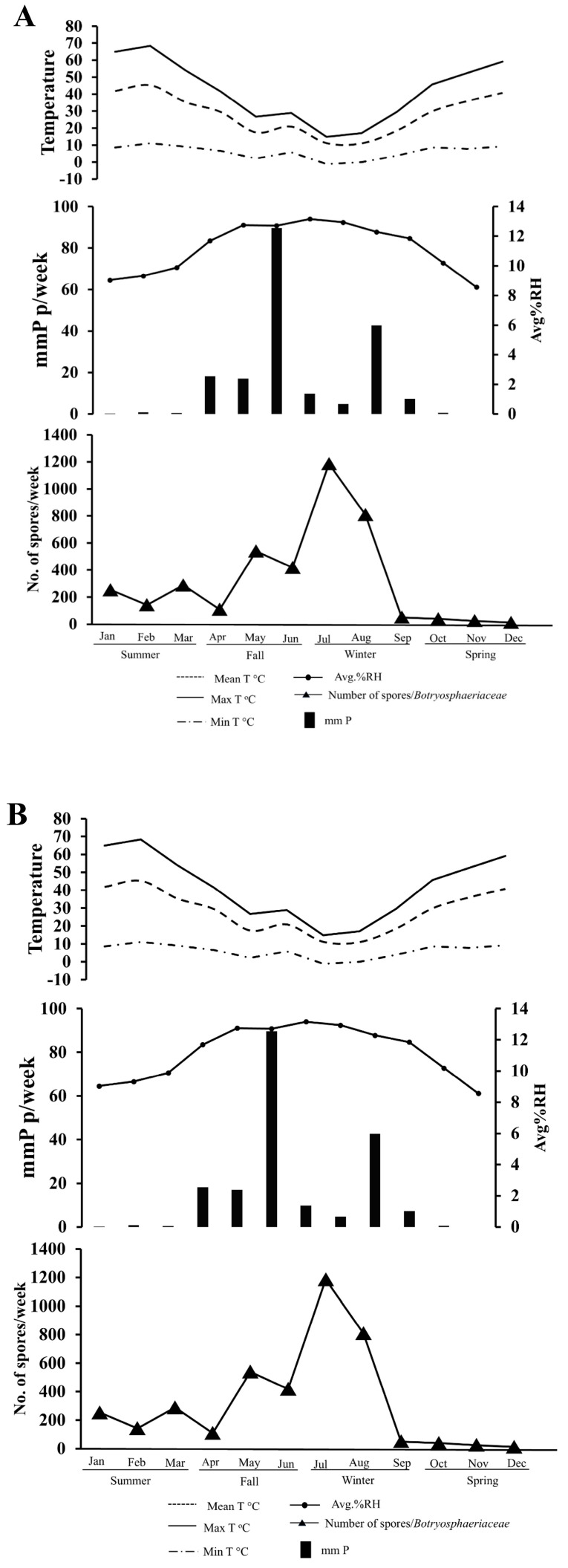
(**A**): Monthly records of minimum, maximum, and average temperatures (T, °C), total rainfall (mm, *p*), average relative humidity (%, RH), and Botryosphaeriaceae conidia counts in 2023 in a walnut ‘Chandler’ orchard located at the San Rafael experimental Station in the Maule Region, Chile. The cardinal temperatures (dashed lines), relative humidity (solid lines), accumulated rainfall (histogram bars), and spore counts (triangles) observed during the monitoring period are shown in the figure. (**B**): Monthly records of minimum, maximum, and average temperatures (T, °C), total rainfall (mm, *p*), average relative humidity (%, RH), and Botryosphaeriaceae conidia counts in 2024 in a walnut ‘Chandler’ orchard located at the San Rafael experimental Station, Maule Region, Chile. The cardinal temperatures (dashed lines), relative humidity (solid lines), accumulated rainfall (histogram bars), and spore counts (triangles) observed during the monitoring period are shown in the figure.

The Maule Region exhibited a typical Mediterranean climate with maximum temperatures peaking in January and declining to minimum values of below 10 °C during the winter months (June–August) in 2023 (Figure 3A). Rainfall patterns were markedly seasonal, with precipitation concentrated from May to August, reaching the highest monthly levels in June, accompanied by relative humidity exceeding 80%. During the dry season (December–March), relative humidity decreased below 50%. This climatic pattern directly influenced Botryosphaeriaceae spore dispersal, with total annual detection reaching 3664 spores. Winter months (July, August, and September) dominated spore release with 1836 spores (50.1%), followed by fall (April, May, and June) with 1054 spores (28.8%). This clear temporal alignment demonstrates that the cold, wet winter conditions of the Mediterranean climate are the primary environmental driver of Botryosphaeriaceae spore release in the region.

**Figure 4:**
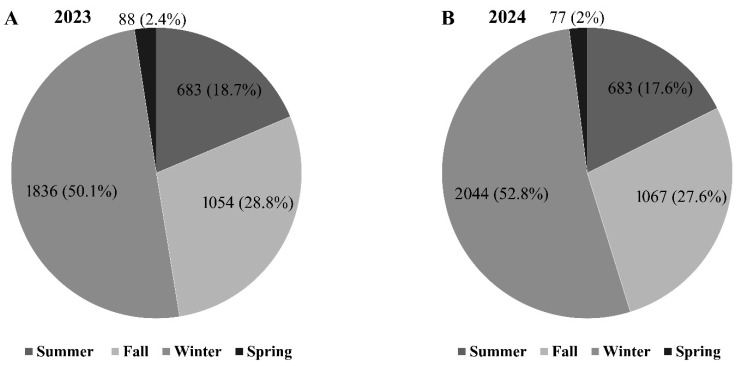
Total accumulated number of Botryosphaeriaceae spores detected in each season during 2023 (**A**) and 2024 (**B**) in a walnut ‘Chandler’ orchard located at the San Rafael experimental Station in Maule Region, Chile.

In comparison, summer (January, February, and March) and spring (October, November, and December) showed minimal activity with 683 spores (18.7%) and 88 spores (2.4%), respectively (Figure 4A). Spore catches remained minimal during hot, dry summer months but increased significantly from April onward, reaching peak abundance in July when precipitation and humidity levels were highest. Pearson correlation analysis revealed that with rainfall (*r* = 0.81, *p* < 0.0001), relative humidity maintained a moderate positive correlation with spore abundance (*r* = 0.51, *p* < 0.05), while maximum temperature showed a significant negative correlation with spore count (*r* = −0.59, *p* < 0.05), demonstrating strong environmental control over conidial dispersal. Temperature parameters exhibited strong intercorrelations, with maximum temperature negatively correlating with relative humidity (*r*= −0.75, *p* < 0.01), reinforcing the inverse relationship between temperature extremes and moisture-dependent spore release. Consequently, the dispersal of Botryosphaeriaceae spores is confined to a narrow seasonal window, primarily dictated by the interplay of high rainfall and humidity coupled with low temperatures.

#### 3.2.2. Climate-Spore Relationship-2024

Similar Mediterranean climatic conditions prevailed, with maximum temperatures peaking in January and minimum temperatures dropping below 5 °C during the coldest months (June–August). Peak rainfall and humidity occurred during June–July, maintaining the seasonal concentration pattern observed in 2024 (Figure 3B). The consistent environmental conditions resulted in increased spore detection, with 3872 total representing a 5.7% increase from the previous year (Figure 3A). Winter continued to dominate spore dispersal with 2044 spores (52.8%), while fall contributed 1067 spores (27.6%), summer 683 spores (17.6%), and spring 77 spores (2.0%) (Figure 4B). The remarkable consistency in both climatic patterns and the resulting spore dispersal between years confirms a stable and predictable seasonal cycle for Botryosphaeriaceae inoculum in this region.

The temporal pattern of spore release mirrored 2023, with low spring activity, increases beginning in May, and peak dispersal occurring in July, coinciding with maximum precipitation periods. Environmental correlations reinforced previous year patterns, with rainfall (*r* = 0.81, *p* < 0.0001), relative humidity maintaining a stronger positive correlation with spore abundance (*r* = 0.61, *p* < 0.05), while maximum temperature demonstrated an intensified negative correlation with spore count (*r* = −0.79, *p* < 0.05). Temperature intercorrelations remained consistent, and relative humidity continued to negatively correlate with temperature extremes, confirming humidity as the primary driver of Botryosphaeriaceae spore dispersal, with over 50% of annual spore release occurring during high-moisture winter months in both study years. The replication of these results across two consecutive years solidifies the model of Botryosphaeriaceae spore release as a highly predictable epidemiological pattern, driven predominantly by the region’s winter humidity.

## 4. Discussion

This study presents the first comprehensive assessment of pruning wound susceptibility to Botryosphaeriaceae and Diaporthaceae species in Chilean walnut orchards, uncovering critical temporal dynamics with direct implications for disease management. Our results indicate that fresh pruning wounds constitute the period of highest vulnerability to infection, with susceptibility diminishing progressively as wounds age. This temporal trend is consistent with findings from other woody hosts—such as almond, apple, and grapevine—where peak susceptibility typically occurs within the first two weeks post-pruning [44,48,50,51,57]. These parallels reinforce the importance of timely wound protection measures in walnut orchards to mitigate pathogen infection during this critical window.

Walnut pruning wounds in our study exhibited the highest susceptibility to infection one day post-pruning, with *D. mutila* inducing the most extensive lesions (51.8 mm in 2023 and 52.5 mm in 2024), followed by *N. parvum* (46.2 mm and 45.2 mm, respectively, as described in Figure 1 and Figure 2). These findings corroborate the aggressive nature of these pathogens, consistent with previous reports identifying *D. mutila* and *N. parvum* as highly virulent on woody hosts such as apple, English walnut, and grapevine [3,21,58].

The progressive reduction in lesion length with increasing wound age observed in our study mirrors trends previously reported in grapevine. For example, Eskalen et al. [57] demonstrated that grapevine canes were most vulnerable to *D. seriata* and *Lasiodiplodia theobromae* within the first two weeks post-pruning, with infection rates declining thereafter. Similarly, our data showed that *D. mutila* lesions in walnut decreased on average from 51.8 mm at 1 day to 36.9 mm at 15 days, 20.3 mm at 30 days, and only 1.4 mm at 45 days post-pruning in 2023, as mentioned in Figure 1. This pattern supports the hypothesis that wound healing mechanisms progressively limit pathogen ingress over time.

Differences in wound susceptibility among fungal species were consistent with prior taxonomic and virulence studies. Wounds remained more susceptible to Botryosphaeriaceae species (*D. mutila*, *N. nonquaesitum*, and *N. parvum*) across all wound ages, whereas *Do. sarmentorum* and Diaporthaceae species exhibited significantly lower virulence. For instance, *Di. australafricana* lesions ranged from 32.4 mm on fresh wounds to 5.9 mm at 45 days, while *Di. patagonica* produced minimal lesions, as shown in Figure 1 and Figure 2. This contrasts with grapevine studies, where certain Diaporthaceae species have shown high aggressiveness [34,59], suggesting that host-specific factors may influence susceptibility and pathogen virulence in walnut. Our findings align with Chen et al. [3] and Jiménez Luna et al. [43], who reported that Diaporthe species are generally less virulent than Botryosphaeriaceae in English walnut.

The presence of Botryosphaeriaceae and Diaporthaceae species in English walnut orchards, previously documented in California [3], is now confirmed in Chilean production systems. Single-species inoculations with Diaporthaceae, particularly *Di. patagonica* resulted in the lowest lesion development across all wound ages. *Dothiorella sarmentorum* exhibited intermediate virulence, with lesion lengths ranging from 26.5 mm on fresh wounds to 4.0 mm at 45 days in 2023, and a slightly higher persistence in 2024 (8.0 mm at 45 days). *Diaporthe foeniculina* showed similar intermediate behavior, with lesions decreasing from 33.3 mm on fresh wounds to 4.3 mm at 45 days in the first year, and to 6.3 mm in the second year outlined in Figure 1 and Figure 2.

The wound healing mechanisms underlying our observed susceptibility patterns are well documented in woody plants. Amponash et al. [60] and Valdez-Tenezaca et al. [44] reported that suberin and lignin deposition begin around grapevine and apple wounds approximately 2 weeks after pruning, creating physical barriers that impede fungal penetration and colonization. Our results showing that walnut pruning wounds can still be infected up to 45 days after cutting, despite a gradual reduction in lesion size, suggest that walnut healing may follow similar timelines but with species-specific variations in the rate of barrier formation. The host genotype has also been reported as a major resistance determinant, and varietal susceptibility differences have been documented in other nut crops, including almonds and pistachio [52].

The significant interaction between pruning wound age × fungal species in both years of trial demonstrated that susceptibility in response to the wound depends on wound age and the specific pathogen involved. These investigations has practical implications, showing that fresh wounds are more susceptible to some fungal pathogens while older wounds are more susceptible to others. Pruning lesions were considerably more susceptible to *D. mutila* than *Di. patagonica*, and even 45 days after pruning, wounds were highly susceptible to *D. mutila* (12.4 mm lesions) with little susceptibility to *Di. patagonica* at the same wound age (2.4 mm lesions) as shown in Figure 1 and Figure 2.

The annual differences in wound susceptibility to some Diaporthaceae and Botryosphaeriaceae species exhibit contrasting patterns, which may indicate that the two families respond differently to the environmental factors influencing wound healing. This year-to-year variability may indicate that environmental factors might influence wound pathogen interactions, as previously described by Serra et al. [61] and Eskalen et al. [57] on the grapevine, where climatic features influence infection risk.

The environment significantly affected the dispersal of Botryosphaeriaceae spores in Chilean walnut orchards, with conidial release being most strongly correlated to rain (*r* = 0.81; *p* < 0.0001) and being the most critical driver of spore release. The relative humidity also had a positively correlated value with the spore abundance (*r* = 0.51 in 2023 and *r* = −0.61 in 2024, *p* < 0.05). On the other hand, the maximum temperature showed a high negative correlation with spore count (*r* = −0.59 in 2023 and *r* = −0.79 in 2024, *p* < 0.05) as cited in Figure 3A,B. These associations are consistent with previous research on Botryosphaeriaceae spore dispersal, where humidity-driven spore release has been observed in Mediterranean climates for a range of hosts, including grapevine and macadamia [62,63].

Our studies seasonal distribution of spore release demonstrated that winter months were the most common period during which spores were detected, with more than half of their total annual quantity (1836 spores/2023 and 2044 spores/2024), as shown in Figure 4A,B. Autumn was the next period most recurrent in spore dispersal, with 28% of the annual spore release (1054 and 1067 spores, respectively). Sporulation was very low in summer and spring, when <20% of the total annual spore release was observed. This seasonal pattern is consistent with Mediterranean climate studies where Botryosphaeriaceae activity peaks were observed during wet seasons [7,43,56,64]. The negative correlation between temperature and spore release observed in our study aligns with findings by Tapia et al. [65], who reported that elevated temperatures during dry periods suppress conidial exudation from pycnidia, while cooler, moist conditions promote spore release.

Rain events were identified as the primary trigger for conidial release, with spores becoming airborne through rain splash and wind-assisted droplets, as previously documented by Amponash et al. [66] and Valencia et al. [64]. The strong correlation between accumulated rainfall and spore counts in our study (*r* = 0.81) suggests that precipitation events serve as the dominant mechanism for spore liberation from mature pycnidia, similar to patterns reported in apple, almond, and pistachio orchards [43,44,67]. The concentration of spore release during the winter months of July and August in our study, when relative humidity exceeded 80% and temperature dropped below 10 °C, coinciding with maximum rainfall, constitutes a period where fresh pruning wounds are exposed to high inoculum pressure coverage to maximize infection risk.

It is well known that species belonging to the Botryosphaeriaceae and Diaporthaceae families develop and survive as saprophytic microorganisms on dead plant tissues, developing fruiting bodies that can produce viable spores. Subsequently, these spores are an important inoculum source to cause branch dieback on English walnut, with pruning wounds being a potential infection court via which spores can enter, infect, and colonize walnut shoots [4,68]. The effect of pruning wounds on infection by fungal trunk pathogens such as Botryosphaeriaceae has already been well demonstrated in Prunus species [47] and grapevine [69,70]. However, to our knowledge, this study is the first to demonstrate the effect of pruning wound age and the susceptibility patterns to both Botryosphaeriaceae and Diaporthaceae species in English walnut under Chilean conditions.

The moderate susceptibility of the Chandler cultivar observed in our study aligns with previous varietal evaluations. López-Moral et al. [52] reported significant varietal differences in susceptibility to Botryosphaeriaceae species among nut crops, including walnut and almonds. The consistent lesion development across both experimental years suggests that Chandler’s susceptibility profile remains stable under similar environmental conditions, providing a reliable baseline for a comparative study with other walnut cultivars.

The information obtained in this work is relevant and determines the temporal dynamics of wound susceptibility to multiple pathogen species in walnut. Our results suggest that special attention should be given to the immediate post-pruning period when wounds are most susceptible, particularly to aggressive Botryosphaeriaceae fungi. Moreover, since wounds remained susceptible for up to 45 days, extended protection strategies may be necessary during high-risk periods coinciding with spore release.

The practical implications for walnut disease management include timing pruning operations to avoid periods of high spore release (winter rainfall periods), implementing protective fungicide applications to fresh wounds, and considering biological control agents for extended protection. Protectant fungicides such as thiophanate-methyl, pyraclostrobin, and tebuconazole have proven effective against Botryosphaeriaceae fungi in grapevine trials [70,71]; their potential in walnut remains to be evaluated. Additionally, biological wound protectants containing *Trichoderma* spp. are emerging as potential alternatives to chemical fungicides [27].

## 5. Conclusions

In summary, pruning wound age and environmental factors could be integrated into the epidemiology of branch canker and dieback in walnut orchards in Chile caused by Botryosphaeriaceae and Diaporthaceae species, as demonstrated in this study. Pruning wound susceptibility in walnut depends on wound age, with 1-day-old wounds showing maximum vulnerability to all tested pathogens, particularly *D. mutila* and *N. parvum*. The progressive decline in susceptibility over 45 days post-pruning provides a clear temporal framework for implementing control studies evaluating chemical and biological agents for protecting wounds to prevent the spread and severity of trunk disease in Chilean walnut orchards. This integration of wound protection protocols, understanding of temporal susceptibility patterns, and knowledge of environmental factors driving spore release should be essential for successfully mitigating canker and trunk disease in walnut production. Future research should include compound testing under field conditions, cultivar resistance level determination, and the establishment of early warning systems through weather-based models and spore trapping networks.

## Data Availability

The raw data supporting the conclusions of this article will be made available by the authors on request.
